# Effects of exosomes of mesenchymal stem cells on cholesterol-induced hepatic fibrogenesis

**DOI:** 10.22038/IJBMS.2023.68858.15003

**Published:** 2023

**Authors:** Mojtaba Rashidi, Emad Matour, Sajad Monjezi, Shahla Asadi Zadeh, Elham Shakerian, Sahar Sabahy, Reza Afarin

**Affiliations:** 1 Cellular and Molecular Research Center, Medical Basic Sciences Research Institute, Ahvaz Jundishapur University of Medical Sciences, Ahvaz, Iran

**Keywords:** Cholesterol, Exosomes, Fibrosis genes, Liver fibrosis, Smad3 protein

## Abstract

**Objective(s):**

Free cholesterol in the diet can cause liver fibrosis by accumulating in Hepatic stellate cells (HSCs). The rate of mortality of this disease is high worldwide and there is no definite remedy for it, but might be treated by anti-fibrotic therapies. MSCs-derived exosomes are known as the new mechanism of cell-to-cell communication, showing that exosomes can be used as a new treatment. In this study, we investigated the ability of exosomes of WJ-MSCs as a new remedy to reduce cholesterol-induced liver fibrosis in the LX2 cell line.

**Materials and Methods:**

MSCs were isolated from Wharton’s jelly of the umbilical cord and the exosomes were extracted. The LX2 cell line was cultured in DMEM medium with 10% FBS, then cells were treated with 75 and 100 μM concentrations of cholesterol for 24 hr. The mRNA expression of TGF-β, αSMA, and collagen1α genes, and the level of Smad3 protein were measured to assess liver fibrosis.

**Results:**

Cholesterol increased the expression of TGF-β, αand -SMA, and collagen1α genes by increasing the phosphorylation of the Smad3 protein. Treatment with Exosomes significantly reduced the expression of TGF-β, α-SMA, and collagen1α genes (fibrosis genes). Treatment with exosomes prevented the activation of HSCs by inhibiting the phosphorylation of the Smad3 protein.

**Conclusion:**

The exosomes of WJ-MSCs can inhibit the TGFβ/Smad3 signaling pathway preventing further activation of HSCs and progression of liver fibrosis. So, the exosomes of WJ-MSCs s could be introduced as a treatment for liver failure.

## Introduction

Liver fibrosis is caused by chronic liver damage that is characterized by excessive accumulation of extracellular matrix (ECM), including collagen (1). The main causes of liver fibrosis include viral hepatitis infections (including hepatitis B and hepatitis C), alcohol abuse, autoimmune diseases, and inherited metabolic disorders (1, 2). Advanced liver fibrosis may progress to cirrhosis, liver failure, hepatocellular cancer (HCC), and death (3, 4). The activation of hepatic stellate cells (HSCs) plays an essential role in the progression of hepatic fibrogenesis (5). HSCs are activated by several factors, like growth factors (including PDGF), inflammatory cytokines (such as IL-1β and TNF-α), chemokines, and oxidative stress products (6, 7). Among the activators of HSCs, transforming growth factor beta-1 (TGF-β1) is a major profibrotic cytokine in the liver. TGF-β1 is critical for the activation of HSCs into myofibroblasts, and then activated HSCs contribute to TGF-β1 production (8, 9) and produce an ECM in the liver that progresses with collagen deposition (6, 7). Another factor is dietary factors that play an important role in the development of liver fibrosis. High dietary cholesterol consumption increases the risk of cirrhosis or liver cancer. Studies have shown that accumulation of cholesterol in the form of free cholesterol in HSCs leads to the activation of HSCs induced by TGF-β, which exacerbates liver fibrosis (10). For this reason, it is important to know the pathological mechanisms associated with this disorder (11). Therefore, it is crucial to identify new anti-fibrotic agents which can prevent the progression of liver fibrosis (5). It has recently been suggested that blockers of the TGF-β/Smad3 signaling pathway could inhibit liver fibrosis in several fibrotic animal models (12, 13).

Human umbilical cord Wharton’s jelly (WJ-MSCs) is a soft matrix inside the umbilical cord around the umbilical arteries and is considered a source of MSCs that can self-renew and differentiate into multiple cells (14). Some studies have shown that the mechanism of MSC tissue damage repair involves paracrine action rather than trans-differentiation (15, 16). Cell-derived exosomes are known as the new mechanism of cell-to-cell communication, showing that exosomes can be used as a new treatment for diseases (15). Exosomes are small extracellular vesicles (EVs), which range from 30 to 120 nm in diameter. These vesicles are secreted by various cells present in many body fluids including blood, urine, cerebrospinal fluid, and saliva. Exosomes-derived MSCs contain proteins, lipids, mRNAs, cytokines, growth factors, and nucleic acids, such as mRNAs and miRNAs (17, 18).

At present, there is little information about the mechanism of exosomes derived from WJ-MSCs in liver fibrosis. In this study, we investigated the effects of exosomes of WJ-MSCs on genes that are involved in liver fibrosis progression, including TGF-β, α- smooth muscle actin (α-SMA), collagen1α genes in the presence or absence of cholesterol in the LX2 cell line (a type of cell derived from HSCs), and also phosphorylation of Smad3 protein level.

## Materials and Methods

***Cultivation and treatment of HSCs***

Cholesterol and RIPA buffer from Sigma, PVDF membranes (Millipore, USA), Fetal bovine serum (FBS) from Gibco, DMEM (Dulbecco’s Modified Eagle Medium), and penicillin and streptomycin antibiotics (Ideazist, IRAN) were purchased. LX-2, an immortalized human HSCs cell line, was given by Professor S. Friedman. First, the LX-2 cells were seeded in 6-well plates (1 × 10^5^ cells per well) in DMEM containing 10% FBS at 37 °C with 5% CO_2_ (19, 20). When cells are approximately 80–90% confluent, the cells must be sub-cultured to ensure proper growth and health of the cells. Second, after 16 hr starvation, the cells were treated with 75 and 100 μM cholesterol and incubated for 24 hr. Next, the exosomes of WJ-MSCs (final concentration of 50 μg/ml) were dissolved in a culture medium and added to the cells for 24 hr. Three groups were considered for the experiment: 1- Control group, 2- Cholesterol treatment group, and 3- Cholesterol treatment group + exosome of WJ-MSCs; after 24 hr, cells were washed twice with PBS, then Real-Time PCR and Western Blots analysis were performed.

***Isolation and culture of WJ-MSCs***

Fresh human umbilical cord was removed from the mothers after full-term cesarean delivery with obtained informed consent. The umbilical cord was washed 3 times in phosphate-buffered saline (PBS) and the umbilical vessels were removed. Wharton jelly was then cut into pieces of 1–3 mm^3^ and placed in a flask with a complete culture medium composed of low glucose-DMEM supplemented with 20% FBS and 100 U/L penicillin-streptomycin. Cord pieces were subsequently incubated at 37 °C with 5% CO_2_. The medium was changed every 3 days after initial plating.

***Diﬀerentiation assays of WJ-MSCs***

WJ-MSCs were cultured in osteogenic and adipogenic differentiation media (BN_0012.4 and BN_0012.5, IRAN), respectively. For osteogenic differentiation, WJ-MSCs were incubated (20,000 cells/ml, 21 days) in osteogenic differentiation media. For adipogenesis, WJ-MSCs were incubated for 3 weeks with adipogenic differentiation media. Finally, the cells were examined using a confocal microscope.

***Detection of WJ-MSCs surface markers***

After the third passage, WJ-MSCs were trypsinized (0.025 % trypsin and 0.02% EDTA). The cells were washed twice with PBS and stained with the following monoclonal antibodies conjugated to various fluoroprobes according to the manufacturer’s recommendations. FITC-conjugated mouse anti-human CD34, FITC-conjugated mouse anti-human CD45, PE-conjugated mouse anti-human CD44, and PE-conjugated mouse anti-human CD105 were needed; all antibodies were purchased from eBioscience Company. The cells were analyzed using BD FACSLyric instrument (Becton Dickinson, San Diego, CA, USA). At least 20,000 events were recorded for each sample, and the results were analyzed using the FlowJo™ software.

***Exosomes isolation***

WJ-MSCs were cultured in a DMEM medium with 15% FBS. Exosomes were separated from the supernatant of the culture by using an EXOCIB isolation kit (CIB Biotech., Iran). Eventually, the isolated exosomes were diluted in 150 µl PBS. The concentration of exosome-related proteins was measured by using bicinchoninic acid (BCA) protein assay kit and was considered equivalent to exosome concentration.

***Characterization of exosome***

Dynamic light scattering (DLS) was performed to analyze the size distribution of the exosomes. For DLS, the exosome content solution was diluted to 1 μg/ml PBS and 0.05% Tween-20, finally the size of them was evaluated by DLS Zetasizer Nano (Malvern Corp, UK) at 23 °C according to the manufacturer’s instructions. The transmission electron microscope (TEM) was used to visualize the exosomes. For TEM, the exosomes were fixed in glutaraldehyde 1% for 20 min. After washing with PBS, they were suspended in distilled water. Then a few drops were placed on the grid and stained with uranyl acetate 1%. Finally, it was evaluated with an electron microscope. 

***Real-time PCR technique***

Total RNA was extracted using an RNA Isolation Kit (Yekta Tajhiz Azma, Iran) according to the manufacturer’s instructions. The complementary DNA (cDNA) was synthesized by using a cDNA synthesis kit (Yekta Tajhiz Azma, Iran). The cDNA templates were amplified using Real-time PCR through the SYBR Green master mix (Ampliqon, Denmark) and QuantStudio™ 3 Real-Time PCR System (ABI Applied Biosystems) following the manufacturer’s instructions.

The following primers were used in RT-PCR: TGF-β:

forward *5’-GTGGACATCAACGGGTTCACT-3’*, reverse: *5’-*

*CTCCGTGGAGCTGAAGCAATA-3’*; αSMA: forward *5’-CCGGGACTAAGACGGGAATC-3’*,reverse*5’-CCATCA*

*CCCCCTGATGTCTG-3’*; *Collagen1α*: forward *5’-GGAATG*

*AAGGGACACAGAGGTT-3’, *reverse *5’-AGTAGCACCATCA*

*TTTCCACGA-3’ *and *GAPDH*: forward *5’-GACAGTCAGCC*

*GCATCTTCT-3’*, reverse *5’-GCCCAATACGACCAAAT*

*CCGT-3’.*

The GAPDH gene was used as an internal control to correct the expression of target genes. 

***Western blotting technique***

HSCs were lysed in RIPA buffer supplemented with protease and phosphatase inhibitors. The BCA method was used to determine the protein concentration. Then, protein samples (30 μg per line) were separated by SDS-PAGE and transferred to PVDF membranes. After that, the membrane was blocked for 2 hr at room temperature in 5% skim milk or bovine serum albumin (BSA) (for p-proteins) in Tris buffer. Antibody detection was performed using an ECL reagent (BioRad) and visualized using a ChemiDoc device (BioRad).

***Statistical analysis***

The results of the experiments with three-time repetitions were analyzed by ANOVA and Tukey’s discriminant test after doing the normality test. The analysis was done using the Parsim 9 software and the significance level for the groups was considered to be less than 0.05.

## Results

***Characterization of WJ-MSCs***

**Figure 1:**
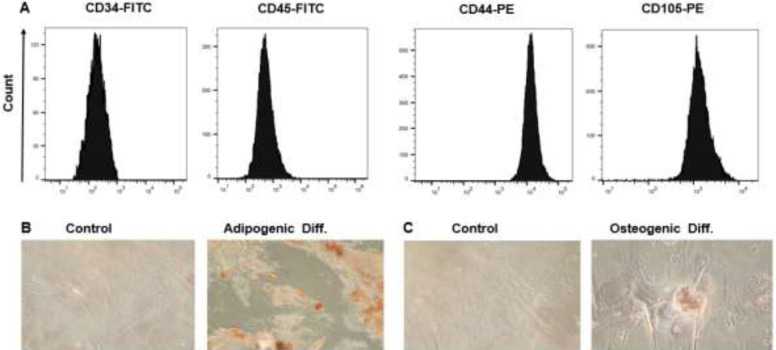
Immunophenotyping and differentiation potentials of WJ-MSCs. (A) Flow cytometry analysis of surface markers showed that WJ-MSC expresses CD44 and CD105 but expresses CD34 and CD45 in a down-regulated manner. (B) Oil Red O staining of WJ-MSCs, intracellular lipid accumulation stained bright red in adipocytes at day 21. (C) Alizarin Red S staining of WJ-MSCs, calcium deposition stained bright orange-red in osteocytes at day 21

To determine the character of WJ-MSCs, we evaluated the expression of surface markers and the potential for laboratory differentiation, as demonstrated by the International Stem Cell Association (21). We used WJ-MSCs isolated from the umbilical cord after the third passage, which are presented as monolayers of spindle-shaped fibroblast-like cells with the ability to adhere to plastic flasks. The expression of WJ-MSCs-specific surface markers was analyzed by Flow cytometry. Flow cytometry analysis of WJ-MSCs showed that the cells were positive for MSC surface markers such as CD44 and CD105, and were negative for monocyte-macrophage (CD45) and hematopoietic stem cell (CD34) markers, which indicated that the isolated cells were MSCs and were not derived from endothelial and hematopoietic cells (Figure 1A). Confirming the potential for *in vitro* differentiation of WJ-MSCs, after 21 days, WJ-MSCs showed numerous lipid droplets visualized by oil red O (Figure 1B). Moreover, when the cells were differentiated into osteoblasts and stained with Alizarin Red, extensive calcium deposition was observed, which is a typical sign of osteogenic differentiation (Figure 1C).

***Exosome characterization***

**Figure 2:**
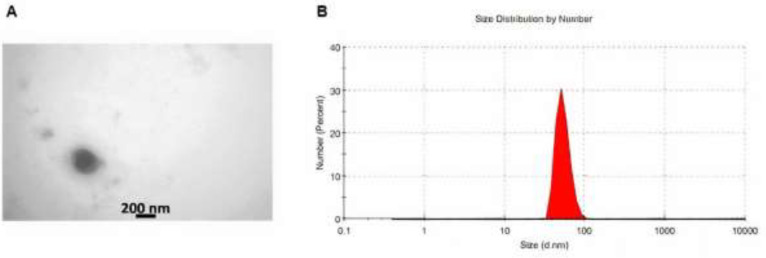
Characterization of exosomes. (A) TEM of the exosomes to visualize the shape and size of these vesicles. (B) Exosome size determination by Malvern zeta sizer. Up to 85% of total exosomes were 73 nm in diameter

The TEM result showed that all exosomes had a spherical shape and range from 50 to 200 nm in diameter (Figure 2A). The size distribution of purified exosomes was measured using a zeta sizer (Malvern Corp.). Up to 85% of the exosomes had a diameter of 73 nm (Figure 2B). 

***Effects of cholesterol and exosome*** ***treatments on gene expression ***

**Figure 3:**
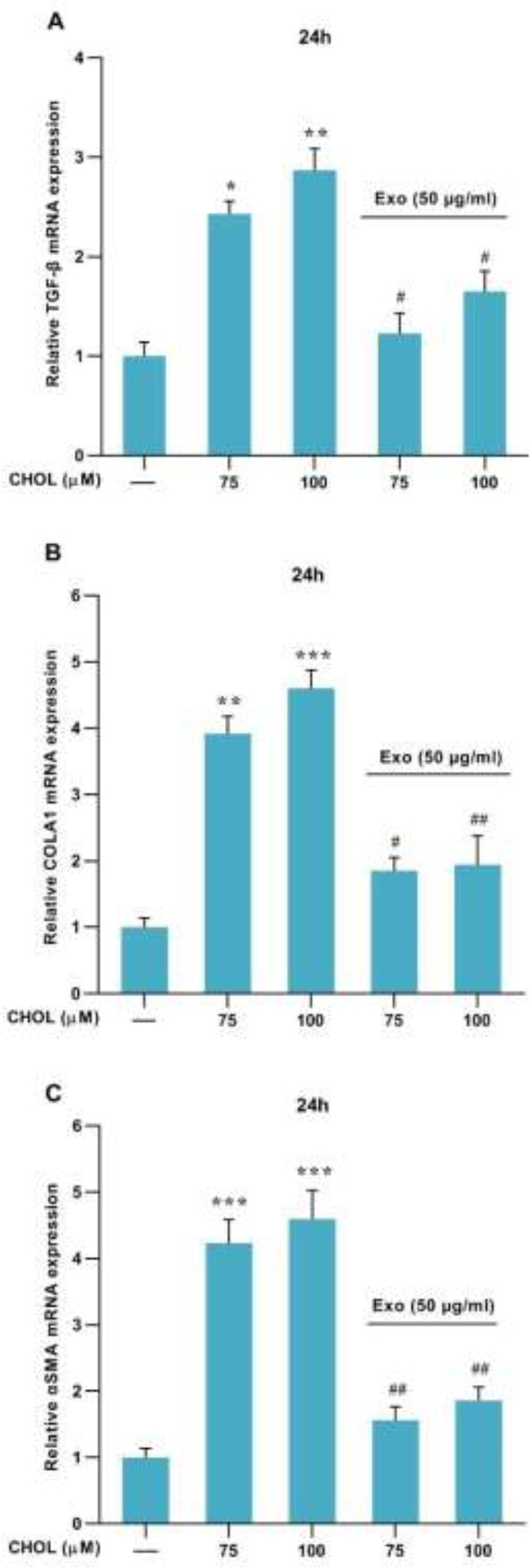
mRNA expression of TGF-β, collagen1α, and αSMA genes in the presence of cholesterol and exosome in LX2 cell line: Results of three replications (Mean±SEM) control have been reported. The significance level was considered *P*<0.05. GAPDH was used as the reference gene (*P<0.05, **P<0.01, ***P<0.001, #*P*<0.05, and ##*P*<0.01)

In treatment with 75 and 100 μM cholesterol concentrations, the Real-Time PCR analyses showed that the mRNA expression levels of TGF-β, α - SMA , and Collagen1α (liver fibrosis marker genes) were significantly up-regulated compared to the control group ( Figure 3 ). The mRNA expression of TGF-β, α-SMA, and collagen 1α was significantly increased as a result of the development of cholesterol-induced liver fibrosis. We also found that exosomes of WJ-MSCs treatment (50 μg /ml) could significantly decrease the level of TGF-β, collagen1α, and α-SMA gene expression in cholesterol-induced liver fibrosis model ( Figure 3A, B, and C ).

***Effects of cholesterol and exosome treatments on Smad3 phosphorylation***

**Figure 4:**
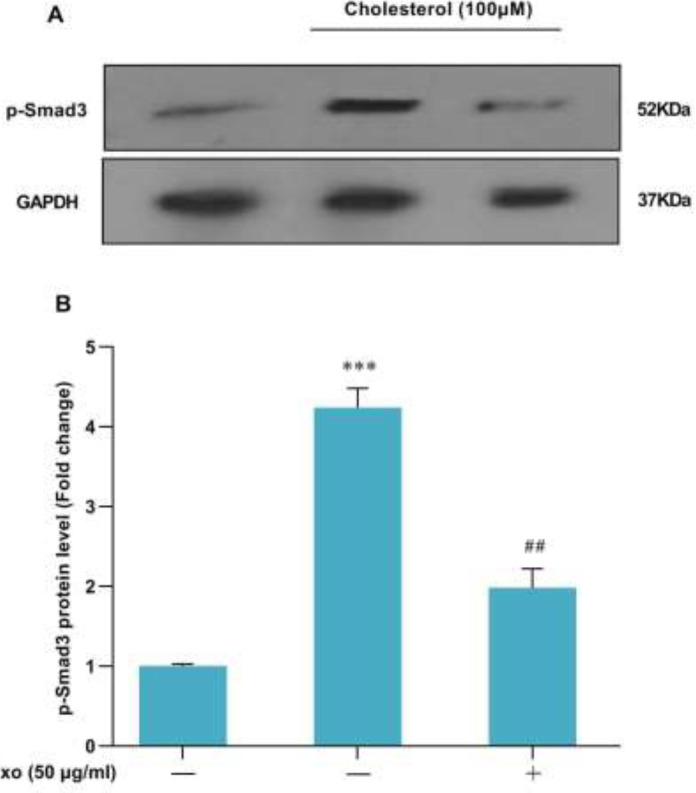
Evaluation of the effect of 4-hour exosome treatment on cholesterol-induced Smad3C phosphorylation in LX2 cell line: Results of three replications (Mean±SEM) were reported. The significance level was considered *P*<0.05. GAPDH protein was used as an internal control gene (***P<0.001, ##*P*<0.01)

To investigate the effects of exosomes of WJ-MSCs on the TGF-β/Smad3 signaling pathway in HSCs, first, the cells were treated with exosomes of WJ-MSCs (50 μg/ml) for 24 hr. The medium was then discarded, and the cells were treated with 100 μM concentration of cholesterol for 24 hr. Following treatment, the P-Smad3 levels were quantified by western blot. The results showed that P-Smab3 levels were increased significantly by treatment of cholesterol (100 μM) vs the control group (Figure 4 A, B). treatment with exosomes of WJ-MSCs improves cholesterol-induced liver cell damage. Significantly, the P-Smad3 levels were down-regulated after 24 hr in the treatment of exosomes of WJ-MSCs in cholesterol-induced HSCs (Figure 4A).

## Discussion

In this study, we studied the exosomes of WJ-MSCs as a treatment in cholesterol-induced liver fibrosis and their effects in regulating HSCs activation and improving liver fibrosis. Liver fibrosis is a reversible process at early stages that is characterized by excessive accumulation of ECM proteins, mainly collagen that eventually leads to liver cirrhosis. HSCs are the main mediator of liver fibrogenesis, also TGF-β1 is the most potent agent for HSCs activation and fibrogenesis (22, 23). TGF-β stimulates ECM in different cell types by activating the signaling pathway. The Smad2/3 protein will be phosphorylated and then stimulates collagen production (24, 25). Therefore, α-SMA is a useful marker for assessing the early stages of hepatic fibrosis (26). The discovery of a new strategy that can target different cellular pathways of liver fibrogenesis is needed for effective treatment (27). It has been well established that hyperlipidemia can cause severe degradation in hepatocytes, eventually leading to hepatic fibrosis. Hypercholesterolemia increased cholesterol and triglyceride levels and affected the fatty acid profile of the liver. hypercholesterolemia is probably responsible for the early stages of fat accumulation in the liver (28). Experimental data showed that in response to hypercholesterolemia, normal liver, and lipid infiltration are disrupted, leading to liver fibrosis in rats (29, 30).

Cell therapy with a multifactorial mechanism of treatment provides an ideal option for current therapy. MSCs are considered a potential candidate for improving liver fibrosis due to their numerous therapeutic effects (27). MSCs are an attractive therapeutic substance in regenerative medicine (31). Several studies have shown the ability of MSCs to improve liver fibrosis and liver function (32, 33). Recently, their paracrine effects have been considered because of the role of factors secreted by MSCs in repairing damaged tissues (31). In this study, we investigated the effects of exosomes derived from WJ-MSCs on cholesterol-activated HSCs in reversing liver fibrosis.

We treated the LX2 cells with cholesterol to activate them and then measured the expression of the TGF-β signaling pathway and liver fibrosis genes. Our data showed that the mRNA expression levels of TGF-β, α-SMA, and Collagen1α were significantly increased after the development of cholesterol-induced liver fibrosis (Figure 3). These results suggest that cholesterol-induced liver fibrosis is related to high TGF-β expression. Regarding the effects of cholesterol expression on HSCs activation, the phosphorylation of P-Smad3 protein was higher in cholesterol treated LX-2 cells. The previous study demonstrated that the consumption of a high-cholesterol diet leads to the accumulation of free cholesterol in HSCs, which suppresses the expression of TGF-β photoreceptor BMP and Bambi, by increasing the TLR4 signaling pathway (11, 34), eventually, the TGF-β signaling pathway increases in HSCs, which activates HSCs and promotes liver fibrosis (11, 35). A high-cholesterol diet exacerbates liver fibrosis, which can lead to increased mRNA expression of TGF-β1 and its signaling pathway in liver tissue (36).

Because the accumulation of free cholesterol in HSCs plays an important role in the sensitization of HSCs to TGF-β1-induced activation and the progression of liver fibrosis (11, 34), we evaluated treatment by exosomes to decrease cholesterol-induced HSCs activation. The LX2 cells were treated with exosomes of WJ-MSCs (50 μg/ml), and the results showed that the expression of TGF-β1, α-SMA, and collagen1α mRNA levels was significantly decreased (Figure 3A, B, and C). We examined the effect of the exosome of WJ-MSC on Smad3 phosphorylation in HSCs treated with cholesterol. In line with these findings, exosomes of WJ-MSCs treatment significantly reduced phosphorylation of Smad3 and TGF-β1 signaling pathways in cholesterol-induced liver fibrosis (Figure 4). These data imply that liver fibrosis had been successfully induced by cholesterol and that the exosome of WJ-MSCs improved the TGF-β1 signaling pathway and liver fibrosis genes. Previous studies have shown that inoculation of exosomes of WJ-MSC in a carbon tetrachloride-induced liver injury model protects liver cells and suppresses liver fibrosis by suppressing epithelial to mesenchymal transition and inactivating the TGF-β1 signaling pathway (15). Exosomes from MSCs can repair tissue damage, inhibit inflammatory responses, and regulate the immune system. They play a crucial role in intercellular communication under physiological conditions and RNA molecules and proteins. The therapeutic use of exosomes as a non-cellular treatment method has several advantages that can be a reasonable justification for replacing it with conventional cell therapy methods. The use of exosomes isolated from stem cell culture supernatant does not have the risks and problems of cell therapy such as aneuploidy and transplant rejection and is more effective than cell culture supernatant and can be a good alternative for treating a variety of diseases (37). Exosomes have advantages over MSCs, including being smaller and less complex than cells, so they are easier to produce and store. Therefore, exosomes can be mentioned as an ideal treatment tool for liver diseases soon (38). 

**Figure 5:**
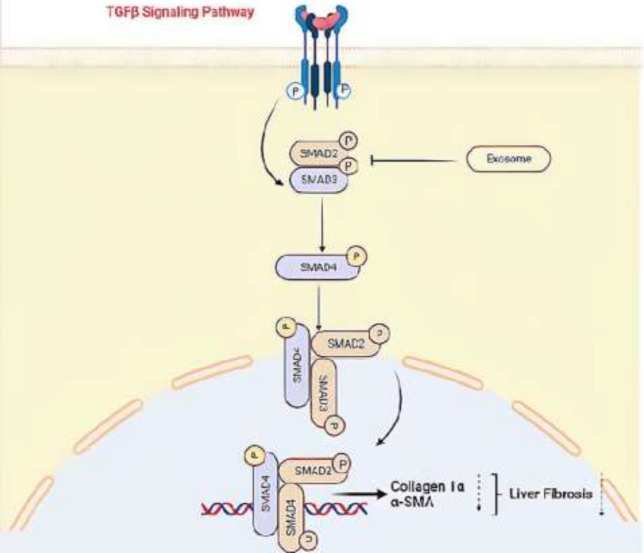
A model of the possible roles of Exosome in TGFβ signaling pathway and liver fibrogenesis

In our study, exosome administration effectively down-regulated the expression of TGF-β, αSMA, and collagen1α and it also reduced the phosphorylation of Smad3 levels in cholesterol-treated liver star cells. Thus, suppression of TGF-β expression can improve hepatic fibrosis, and its mechanism of action is through the TGF-β/Smad3 signaling pathway (Figure 5). This study is useful to develop effective therapies for exosomes of WJ-MSCs. The findings of this study showed that focus on exosomes of WJ-MSCs in cholesterol-induced HSCs is the target of new therapeutic strategies for the treatment of liver fibrosis. Our research was done in cell lines so we suggest doing research *in vivo*. More studies are needed to evaluate the antifibrotic effects of exosomes of WJ-MSCs to treat patients with liver fibrosis and cirrhosis.

## Conclusion

Cholesterol accumulation in HSCs can activate them and increase fibrosis gene expression, leading to the progression of liver fibrosis. We found that the exosomes of WJ-MSCs have the ability to reduce the expression of fibrosis genes so the exosome of WJ-MSCs can be introduced as a safe remedy for liver fibrosis. All mechanisms are regulated by the TGF-β1 signaling pathway, which has an important role in liver fibrosis.

## Authors’ Contributions

RA and MR designed the study. RA performed all assays. EM analyzed the data. SM wrote the first draft. SHA and ESH revised the manuscript. SS contributed to interpreting the results. All authors read and approved the final manuscript.

## Conflicts of Interest

The authors have no conflicting financial interests.
